# Measuring impact: a cross-sectional multi-stage cluster survey to assess the attainment of durable solutions in post-tsunami Aceh, Indonesia

**DOI:** 10.1186/1471-2458-14-1168

**Published:** 2014-11-17

**Authors:** Christopher Lee, Shannon Doocy, Anwar Deli, Thomas Kirsch, William Weiss, Courtland Robinson

**Affiliations:** Center for Refugee and Disaster Response, Johns Hopkins Bloomberg School of Public Health, Baltimore, MD USA; Department of Internal Medicine, University of California – San Francisco, San Francisco General Hospital, 505 Parnassus Ave Rm 987, San Francisco, CA 94143 USA; Syiah Kuala University, Banda Aceh, Indonesia

**Keywords:** Disaster, Impact evaluation, Tsunami, Humanitarian assistance, Displacement, Human rights, Durable solutions

## Abstract

**Background:**

There exists little agreement on the choice of indicators to be used to assess the impact of humanitarian assistance. The 2004 Indian Ocean tsunami led to significant mortality and displacement in Aceh Province, Indonesia, as well as a nearly unprecedented humanitarian response. Six years after the disaster we conducted an impact assessment of humanitarian services rendered in Aceh using a comprehensive set of rights-based indicators and sought to determine modifiable predictors of improved outcomes in disaster-affected households.

**Methods:**

A sample of 597 returned and non-returned households in Banda Aceh and Meulaboh was selected using a multistage stratified cluster survey design. We employed principle components analysis and the *Framework on Durable Solutions for Internally Displaced Persons* to develop a comprehensive and rights-based approach to humanitarian impact measurement using multivariate regression models.

**Results:**

The attainment of durable solutions was equivalent in both returned households 100.1 [CI] 97.63-102.5) and households that integrated elsewhere (99.37 [CI] 95.43-103.3, *P* = 0.781). Standard of living as well as education and health facility satisfaction increased significantly whereas monthly income decreased after the tsunami, from 2585241 IDR ([CI] 2357202–2813279 IDR) to 2038963 ([CI] 1786627–2291298 IDR, *P* < 0.001). Shelter (*P* = 0.007) and legal assistance (*P* < 0.001) were both significantly associated with positive durable solutions outcomes, whereas prolonged displacement duration was significantly associated with poorer outcomes (*P* < 0.001). Livelihood assistance received after one year was associated with higher odds of increasing or maintaining pre-tsunami income levels (OR = 3.02, *P* = 0.008), whereas livelihood assistance received within one year was associated with lower odds of attaining pre-tsunami income (OR = 0.52, *P* = 0.010).

**Conclusions:**

We find that after adjusting for pre-tsunami conditions and tsunami-related damages, the impact of sectoral responses can be assessed. The duration of displacement was the strongest negative predictive factor for the attainment of durable solutions, suggesting that measures to reduce displacement time may be effective in mitigating the long-term effects of disaster on households. The durable solutions framework is a novel and effective impact measurement tool and can be used to identify factors amenable to intervention and inform future disaster recovery efforts.

**Electronic supplementary material:**

The online version of this article (doi:10.1186/1471-2458-14-1168) contains supplementary material, which is available to authorized users.

## Background

On December 26, 2004, an earthquake measuring 9.2 on the Richter scale off the west coast of northern Sumatra caused a tsunami leading to an estimated 128,645 deaths and over 500,000 internally displaced in Aceh Province, Indonesia [1]. Within one year of the disaster, 14% of the population of Aceh continued to be internally displaced, although that number decreased to less than 0.1% by 2008 [2]. Despite the diminished numbers of the displaced and numerous evaluations of humanitarian assistance in the post-tsunami context [2, 3], little is known about which aspects of the relief and recovery process were most beneficial at the household level.

Two barriers to evaluating humanitarian effectiveness are (1) the lack of coherent and modifiable predictors that may be used to assess particular components of successful (or unsuccessful) interventions, and (2) inadequate outcome measurement or definition. Outcomes and predictor variables thus need to be appropriately selected prior to study conception in order to “show that observed changes are caused by a particular programme or activity rather than by other factors” [4].

Selection of predictor covariates in the aftermath of disaster has been problematic. This debate has been articulated most clearly in studies of mental health, wherein the trauma-focused model has largely been supplanted by the psychosocial model, which emphasizes the role of everyday stressors and destruction of social networks. Despite the dose-effect relationship between traumatic events and the development of post-traumatic stress disorder (PTSD) [5], models accounting for only traumatic covariates explain relatively little of the variance in PTSD [6]. Importantly, models accounting only for traumatic experiences at the time of disaster or displacement inadequately account for post-disaster effects, including aspects of the humanitarian response and household recovery. Notably, these are the very covariates required to determine whether there are aspects of the recovery effort that may be intervened upon in future disaster to improve outcomes.

With respect to outcomes, Bolton et al. concluded that, “the effectiveness of humanitarian programs normally is evaluated according to a limited number of pre-defined objectives… and as such, are inadequate benchmarks for understanding the overall effectiveness of aid” [7]. The need for comprehensive indicators in recovery assessment has been echoed by Arlikatti et al. [8] and Martin et al. [9]. To address these issues, focus has turned to impact assessment, which measures the “positive and negative, primary and secondary long-term effects produced by a development intervention, directly or indirectly, intended or unintended” [10] and in the humanitarian context, should “focus on the end of the results chain” [11]. The Active Learning Network for Accountability and Performance in Humanitarian Action (ALNAP), further emphasizes the importance of impact assessment taking into account the needs and outcomes as they pertain to beneficiaries and local actors [4].

Increasingly in disasters and conflicts producing internally displaced persons (IDPs), the concept of ‘durable solutions’ has been applied as in the refugee literature. A durable solution is reached when an internally displaced person returns to his or her place of origin, locally integrates into the village they were displaced to, or resettles in another part of the country – thus marking “the end of displacement” [9]. In order to determine when such a durable solution has been reached, the UN General Assembly accepted the *Framework on Durable Solutions for Internally Displaced Persons* in 2010 which recognizes that “the displaced — whether they return to their homes, settle elsewhere in the country or try to integrate locally — usually face continuing problems, requiring support until they achieve a durable solution to their displacement” [12]. The *Framework* is inherently tied to international refugee, human rights, and humanitarian law by its linkages to the *Guiding Principles for Internal Displacement* [13]. With respect to outcomes measurement, the *Framework* provides indicators to assess whether durable solutions have been achieved, including adequate standard of living, safety and security, access to livelihoods, justice and reparation, restitution of property, recovery of documents, family reunification, and participation in all levels of public affairs. Using durable solutions as a framework thus allows for the application of a coherent set of international normative and rights-based measures across multiple domains to assess humanitarian impact.

In this study, we operationalized the *Framework on Durable Solutions* with two primary aims. Our first aim was to assess the degree to which durable solutions have been attained in Aceh Province following the tsunami using five key criteria from the *Framework* that applied specifically to communities surviving natural disasters: adequate standard of living, safety and security, participation in public affairs, access to livelihoods, and restitution of housing and property. Using these indicators, we created a multivariate measure of the attainment of durable solutions for each household by principle components analysis (PCA). The creation of the durable solutions score allowed us to identify predictors of successful durable solutions outcomes by creating models to measure not only pre-tsunami conditions and tsunami-related damages but also elements of displacement and humanitarian assistance that may be salient to policy-making in future large-scale disaster recovery programs. Here we find that the use of durable solutions to measure outcomes offers a coherent set of human rights-based indicators that are readily implemented in a household survey format.

## Methods

### Survey design

**Figure 1:**
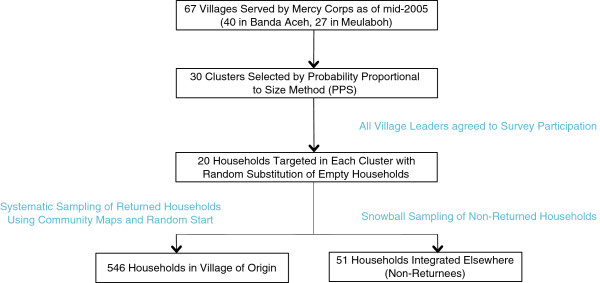
**Sampling strategy.**

The study employed a multistage cluster sample survey design. Two previous surveys of the same study population had been conducted in 2005 and 2006 with an international non-governmental organization, and the cluster selection procedures were preserved across surveys in order to examine trends of the recovery process, although only the data from this round of sampling is presented here. The study population comprised an estimated population of 20,000 households within 67 villages served by the organization in proximity to two cities in Aceh Province: Banda Aceh and Meulaboh (Figure 1). Clusters were selected from these two areas using the probability proportional to size (PPS) method to create a self-weighted sample. For a sampling frame in Round 1, estimates of the number of surviving households in all 67 villages were based on reports obtained from local officials in July 2005. For the 40 villages in Banda Aceh, the number of surviving households was estimated at 10,421 and for the 27 villages in Meulaboh, the number of surviving households was estimated at 8,717. Using a random start number and the appropriate sampling interval, 16 clusters in 13 villages were selected in the Banda Aceh area and 14 clusters in 12 villages were selected in the Meulaboh area.

The sample size was preserved from the first round of survey data collection in 2005. At that time, the proportion of key characteristics of interest (for example, percentage returned or displaced) was presumed to be 50%. The margin of error was set at ±5% and the design effect for cluster sampling was assumed to be 2. These inputs yielded a sample size of 534, which was increased to 600 to allow for non-response and incomplete surveys. Given the selection of *n* = 30 clusters, we selected *m* = 20 households per cluster, which yielded 600 households. The sample size in this cross-sectional analysis was adequate to detect key differences between pre-tsunami conditions and current conditions as well as between those who had returned to their village of origin and those who had integrated elsewhere. The criteria for inclusion were age 18 years or older and ability to participate in the interview and provide informed consent. The exclusion criterion was not having been a resident in the survey cluster prior to the tsunami.

Sampling at the household level was conducted after obtaining permission from the local village authorities in addition to being provided an updated map of the village with a sampling frame and an estimate of the number of pre-tsunami households who had permanently relocated elsewhere. We defined a household as a “group of people who normally lived under the same roof and shared meals” [14]. The village maps provided by the authorities were used to assist with sub-village and household sampling procedures, and systematic random sampling within sub-villages was undertaken with households sampled being proportionate to the sub-village size, using a start number determined *a priori* using a random number generator. Households who had not returned to the village of origin were sampled using a non-probability snowball methodology, whereby current village residents undertaking the survey provided contact details and place of residence of non-returnees. A random subsample of the referred households from the original cluster was then actively traced to their new location of residence and sampled there. Although the non-returnees do not constitute a random sample, we did not have alternate means in which to trace previous residents of a village of origin and capture the diversity of post-migration villages of residence. For instance, had we sampled a village known to have a high proportion of relocated households and traced these residents back to their original villages of origin, not only would we have found very few who had originated from our clusters under study, but their living conditions may have systematically differed from those households who had moved to a village with a lower proportion of relocated households. Although numbers of non-returned households were ascertained from village leaders, the mean percentage of non-returnees was low at 4.41% (SD 6.39%), so non-returned households were oversampled 2:1 to represent 8.54% of the study sample.

For the purposes of local recruitment and ethical review, we partnered with Syiah Kuala University in Banda Aceh, Indonesia. This study was approved by the Institutional Review Board of the Johns Hopkins Bloomberg School of Public Health and by the Ethical Review Committee at Syiah Kuala University. The data used contained no personal identifiers of study participants, and participants provided oral informed consent. No participants refused consent or participation in the study.

### Questionnaire design

The questionnaire was intended to capture pre-tsunami living conditions, tsunami-related damages, the pattern and timeline of displacement and assistance received from the time of the tsunami, and current household conditions. Pre-tsunami living conditions were ascertained from retrospective recall, and items assessed were preserved from previous study rounds. We found no significant differences between the mean household sizes and reported tsunami deaths between the three study rounds (from 2005 and 2006), which provided assurance that the recall was accurate at this later time (data not shown). Measures of household tsunami impact were adapted from those applied by Irmansyah et al. [15]. We tracked displacement of the household using a matrix encompassing the location of displacement, proximity from original home (by participant’s estimate), duration of the displacement (in months), housing type, and reason for leaving. Satisfaction measures with community infrastructure and participation in public affairs were measured on a five-point Likert scale that was accompanied by a visual analogue scale with culturally appropriate visual anchors to which respondents pointed. The completed English form of the questionnaire was adapted with input from the Ethical Review Committee of Syiah Kuala University, and was translated into Bahasa Indonesia by a translator, and independently back-translated into English. The receipt of assistance from local and international actors was ascertained by recall and was coded as a binary variable (1 for yes, 0 for no). The sectors of shelter, livelihoods, food, drinking water, water and sanitation, healthcare, and legal assistance were documented as received at three time points: within 1–2 months of the tsunami, within 1 year of the tsunami, or within 6 years of the tsunami (by the time of survey administration). Livelihood assistance reflected the DFID accepted definition: “a livelihood comprises the capabilities, assets (stores, resources, claims and access) and activities required for a means of living” [16]. Freedom of movement was measured using a categorical response to feeling unsafe traveling to public or community spaces, homes of friends or family members, local markets, or fields around the village. The variable was recoded as a binary outcome if the respondent answered ‘yes’ to any of the categorical responses [17].

### Data analysis

All data analysis was performed using Stata version 11 (StataCorp, College Station, TX), adjusting for stratification and clustering in the sampling procedure to generate a robust Taylor linearized standard error for calculation of confidence intervals and *P* values for regression coefficients. Comparisons of returnees and non-returnees as well as pre-and-post tsunami conditions were performed by Wald testing after calculating robust standard errors (for continuous variables) or Rao-Scott significant tests for test of equivalence of proportions.

#### Principal components analysis

In order to generate a summary score for the five selected indicators for the attainment of durable solutions (adequate standard of living, safety and security, access to livelihoods, participation in public affairs, and restitution of property), and given the lack of meaningful weightings for the various items contributing to each indicator, we undertook principal components analysis, a method used increasingly often to generate self-weighted socio-economic indices [18–20] as well as to generate scores examining several unique indices to assess programmatic impact on a community [21].

#### Regression analyses

We performed multiple linear regression analysis of the durable solutions index total score in a hierarchical manner, with the final model selected using the coefficient of determination. Model fits were assessed assuming simple random sampling, and after selected, were re-run using design based analysis with Taylor linearized standard errors. The final model applied to the durable solutions total index score was then applied to each of the durable solutions indicator scores in order to understand the unique contributions of covariates (including pre-tsunami conditions, tsunami damages, displacement, and assistance received, adjusted for pre-tsunami household and community conditions) to the indicator scores. Multiple logistic regression was performed by dichotomizing the outcome (positive change in monthly household income compared to inflation-adjusted pre-tsunami income, using the World Bank estimate for inflation rate in Aceh). The final model was selected using Akaike’s Information Criterion (AIC).

## Results

### Participants

A total of 597 households were included in the analysis, representing both households that had returned to their original villages of origin as well as those households originally from the sampling cluster but that had since moved elsewhere. The characteristics of those who had integrated elsewhere versus those who had returned to their villages of origin were similar with respect to sex, although returnee respondents were significantly older (41.36 v 38.06 years, *P* = 0.005) (Table 1). Although levels of tsunami-related damage were not significantly different between the two groups, those that integrated elsewhere were significantly less likely to have owned a home prior to the tsunami (39.22% v 97.62%, *P* < 0.001) and were displaced for significantly longer before residing in their current location (29.71 v. 19.80 months, *P* = 0.002). There was no significant difference in the number of tsunami deaths per household; the average for the entire sample was 0.98 deaths per household (0.61 – 1.35).

**Table 1:** **Participant characteristics, by return status**

	Integrated elsewhere (n = 51) mean (95% CI)	Returned to village of origin (n = 546) mean (95% CI)	***P***
Age of Respondent	38.06 (35.94 – 40.18)	41.36 (40.39 – 42.34)	0.005
Sex			
Male	39.22%	30.16%	
Female	60.78%	69.84%	0.273
Pre-Tsunami Household Size	5.39 (4.97 – 5.81)	5.47 (5.24 – 5.69)	0.703
Home Owned Before Tsunami	39.22% (20.32% – 58.11%)	97.62% (95.88% – 99.36%)	<0.001
Distance to Coast (km)	2.32 (1.55 – 3.08)	3.82 (1.99 – 5.65)	0.144
Number of Tsunami Deaths per Household	0.84 (0.46 – 1.23)	1.00 (0.62 – 1.38)	0.311
Level of Household Tsunami Damage			
None	0.00	3.66%	
Minor (Habitable)	5.88%	19.38%	
Major (Uninhabitable)	37.25%	36.01%	
Destroyed Completely	56.86%	40.95%	0.332
Total Displacement Duration (months)	29.71 (25.01 – 34.40)	19.80 (17.22 – 22.38)	0.002

### Pre- vs post-tsunami indicators

Using the *Framework on Durable Solutions for Internally Displaced Persons* to develop appropriate measures for adequate standard of living, safety and security, access to employment and livelihoods, participation in public affairs, and restitution of housing, land and property, we sought to compare current conditions to those immediately prior to the 2004 tsunami (Table 2).

**Table 2:** **Current conditions compared to pre-tsunami conditions**

	25 December 2004	Currently	***P***
Adequate standard of living			
Number of Rooms	5.88 (5.74 – 6.02)	5.36 (5.24 – 5.49)	0.000
Adequate Space*	0.95 (0.93 – 0.96)	0.93 (0.91 – 0.95)	0.334
Type of Roof (%)			
Leaves	7.33 (4.80 – 9.87)	0.50 (-0.24 – 1.24)	0.000
Metal	89.83 (87.07 – 92.60)	97.00 (94.91 – 99.09)	0.000
Tile	2.50 (0.56 – 4.44)	2.17 (0.45 – 3.88)	0.590
Type of Walls (%)			
Wood/Bamboo	31.17 (21.99 – 40.35)	4.83 (0.65 – 9.02)	0.000
Brick/Concrete	66.83 (57.45 – 76.22)	91.50 (86.69 – 96.31)	0.000
Main Water Source (%)			
Delivered	0.50 (-0.54 – 1.55)	2.83 (0.59 – 5.07)	0.072
Well Water	82.83 (73.62 – 92.05)	56.83 (46.26 – 67.41)	0.000
Piped Water	15.33 (6.22 – 24.44)	39.17 (27.83 – 50.50)	0.000
Toilet Location (%)			
Public/Shared	2.33 (1.23 – 3.44)	0.67 (0.03 – 1.31)	0.004
Outside, Private	27.67 (18.66 – 36.67)	4.33 (1.86 – 6.81)	0.000
Inside, Private	67.67 (57.84 – 77.50)	94.00 (90.98 – 97.02)	0.000
Income Spent on Food (%)	70.43 (68.79 – 72.06)	73.84 (72.24 – 75.44)	0.000
School Available for Child to Attend (%)	99.00 (97.92 – 100.08)	98.83 (97.29 – 99.36)	0.166
Satisfaction with School Services^+^	3.80 (3.73 – 3.87)	4.33 (4.27 – 4.39)	0.000
Satisfaction with Local Health Services^+^	3.69 (3.63 – 3.76)	4.11 (4.07 – 4.16)	0.000
Satisfaction with the Way Health Care Runs in the Country^+^	3.81 (3.74 – 3.87)	4.20 (4.16 – 4.25)	0.000
Satisfaction with Water Services^+^	3.90 (3.84 – 3.97)	3.79 (3.70 – 3.87)	0.050
**Safety, Security and Freedom of Movement**			
Perceived Safety in the Community^+^	4.08 (3.93 – 4.23 )	4.63 (4.57 – 4.68)	0.000
Experienced an Act of Violence in past 5 years (%)	3.01 (1.38 – 4.64)	0.33 (-0.15 – 0.82)	0.004
Any Restriction on Freedom of Movement over 5 year Period (%)	24.07 (18.93 – 29.22)	2.02 (0.76 – 3.28)	0.000
**Access to Employment and Livelihoods**			
Proportion of Household Members 16–65 Employed (%)	51.94 (48.92 – 54.95)	50.24 (47.48 – 52.99)	0.355
Head of Household Employed (%)	99.29 (98.61 – 99.97)	96.27 (94.52 – 98.02)	0.001
Total Monthly Income (Rp, inflation-adjusted)	2585241 (2357202–2813279)	2038963 (1786627–2291298)	0.000
**Participation in Public Affairs**			
Community Decision Making^+^	4.12 (4.05 – 4.18)	4.12 (4.04 – 4.19)	1.000
Government Responsiveness^+^	3.69 (3.61 – 3.76)	3.76 (3.67 – 3.83)	0.110
**Restitution of Housing, Land and Property**			
Home Owned (%)	92.62 (88.80 – 96.43)	98.66 (97.71 – 99.61)	0.002
Living on Same Plot (%)		79.26 (74.63 – 83.89)	

^+^Satisfaction measure on a likert scale from 1 (Very Dissatisfied) to 5 (Very Satisfied).

Although there was a significant decrease in the number of rooms per house after the tsunami (5.88 v 5.36, *P* < 0.001), the quality of housing significantly increased. After the tsunami (as of 2011) significantly fewer homes had leaf roofing (0.50% v 7.33%, *P* < 0.001) and significantly greater homes had roofs made from metal (97.00% v 89.83%, *P* < 0.001). Similarly, there was a significant improvement in the materials used to construct the housing, as 91.50% were made of brick or concrete after the tsunami versus 66.83% made of brick prior to the tsunami (*P* < 0.001). Water and sanitation facilities also significantly improved, with 94.00% of homes having a private indoor toilet currently versus 67.67% in 2004 (*P* < 0.001) concomitant with an increase in the number of homes with piped water services (39.17% post-tsunami v 15.33% pre-tsunami, *P* < 0.001). While there was no significant change in the number of households with access to schools for school-aged children (99% both pre- and post-tsunami), the satisfaction with school services significantly increased, as did the perceived satisfaction with both local healthcare services and national healthcare services (all *P* values <0.001).

All measures of safety and security have improved since the time of the tsunami. Only 0.33% of the sample experienced any act of violence or kidnapping in the five years since the tsunami, compared to 3.01% who had experienced an act of violence or kidnapping in the five years prior to the tsunami (*P* = 0.004). Participants’ perceived safety in the community significantly increased after the tsunami (*P* < 0.001). The number of participants reporting any restriction on their freedom of movement decreased dramatically (24.07% prior to the tsunami v 2.02% at the time of interview, *P* < 0.001).

Whereas most measures of standard of living have shown improvement after the tsunami, markers of household wealth decreased. After adjusting for inflation, household incomes significantly decreased approximately 20% after the tsunami (2,585.241 IDR v. 2,038,963 *P* < 0.001). There was a concomitant increase in the proportion of household income that was spent on food at the time of the interview compared to prior to the tsunami (70.43% prior to the tsunami v 73.84% at the time of interview *P* < 0.001), reflecting fewer opportunities for savings and non-essential expenditures. There was also a marginal but statistically significant decrease in the employment status of heads of households (99.29% v 96.27%, *P* < 0.001), although the proportion of household members employed did not significantly change. There were no significant changes in respondents’ perception of government responsiveness to community inputs, and home ownership significantly increased in the period following the tsunami (from 92.62% to 98.66%, *P* = 0.002).

### Predictors of durable solutions

In order to determine predictors of the successful attainment of durable solutions, we created a composite index score using principal components analysis on each of the domains measured in Table 2. This composite score was regressed as an outcome measure on predictor covariates including pre-tsunami conditions, tsunami damages, displacement experiences, and assistance received (Table 3). In the final multivariate linear regression model, after adjusting for pre-tsunami household and community infrastructure conditions, we found that positive pre-tsunami predictors of successful durable solutions outcomes were larger household sizes, college or university education of the head of household (compared to heads of household with less than primary education), the number of household members employed before the tsunami, and the pre-tsunami household monthly income. Conversely, tsunami damages were negative predictors of successful outcomes; higher numbers of tsunami deaths and higher levels of damage (such that the household was uninhabitable) were significantly negatively associated with the durable solutions index score.

**Table 3:** **Durable solutions total index**

	Model A	Model B	Model C	Model D ^+^
**Pre-Tsunami Conditions**				
Household Size	0.314 (-0.251 – 0.879)	0.813*** (0.265 – 1.361)	0.847*** (0.314 – 1.379)	0.632** (0.0760 – 1.187)
Education of Head of Household				
Less than Primary	REF	REF	REF	REF
Primary School	-0.483 (-3.590 – 2.624)	0.166 (-2.635 – 2.968)	0.978 (-1.704 – 3.661)	0.0894 (-3.053 – 3.232)
Secondary School	0.255 (-2.744 – 3.253)	0.73 (-1.941 – 3.400)	1.554 (-1.065 – 4.173)	0.00131 (-2.752 – 2.755)
High School	1.858 (-1.482 – 5.198)	2.198 (-0.809 – 5.205)	2.595* (-0.292 – 5.481)	1.215 (-1.718 – 4.148)
College or University	8.819*** (4.631 – 13.01)	8.202*** (5.006 – 11.40)	8.121*** (5.257 – 10.99)	5.697*** (2.816 – 8.578)
Number of Household Members Employed	3.058*** (1.672 – 4.444)	2.911*** (1.440 – 4.383)	3.134*** (1.592 – 4.676)	2.746*** (1.080 – 4.412)
Employment Status of Head of Household	-2.644** (–4.982 – 0.307)	-4.047*** (-6.326 – 1.769)	-1.972 (-4.679 – 0.736)	3.714 (-1.112 – 8.540)
Home Owned	-0.104 (-3.018 – 2.811)	-0.56 (-3.640 – 2.519)	-0.628 (-4.901 – 3.644)	0.226 (-3.610 – 4.061)
Monthly Income ($10 USD)	0.280*** (0.196 – 0.363)	0.260*** (0.178 – 0.342)	0.224*** (0.146 – 0.302)	0.234*** (0.152 – 0.317)
**Tsunami Damages**				
Distance of Home from Coast (km)		-0.008** (-0.014 – –0.002)	-0.003 (-0.009 – 0.004)	0.001 (-0.007 – 0.009)
Number of Tsunami-related Deaths in Household		-1.188*** (–1.822 – 0.553)	-1.286*** (–1.862 – 0.709)	-1.091*** (–1.718 – –0.464)
Tsunami–Related Damage				
No Damage		REF	REF	REF
Minor Damage		2.273 (-1.788 – 6.333)	0.663 (-3.061 – 4.387)	-1.579 (-3.637 – 0.479)
Major Damage		-1.895 (-7.333 – 3.543)	-2.008 (-6.597 – 2.581)	-3.552** (-6.880 – 0.223)
Destroyed Completely		-0.542 (-6.522 – 5.438)	-0.453 (-5.492 – 4.587)	-3.164* (-6.602 – 0.274)
**Displacement**				
Living in Same Village			-0.355 (-3.881 – 3.171)	-1.387 (-4.901 – 2.128)
Total Displacement Duration (months)			-0.125*** (–0.201 – 0.049)	-0.113*** (–0.165 – 0.061)
Ever Displaced to a Barrack			0.174 (-1.690 – 2.037)	0.521 (-1.179 – 2.222)
Ever Stayed with a Host Family			1.154 (-0.407 – 2.715)	0.94 (-0.544 – 2.424)
**Assistance Received**				
Received Shelter Assistance				2.695*** (0.828 – 4.563)
Received Livelihood Assistance				-1.065 (-2.516 – 0.386)
Received Food Assistance				-8.562*** (–14.85 – –2.269)
Received Drinking Water Assistance				3.276 (-1.012 – 7.564)
Received Water for Hygiene				-2.859 (-6.762 – 1.045)
Received Healthcare Assistance				3.024* (-0.578 – 6.627)
Received Legal Assistance				2.356*** (1.179 – 3.532)
Received Sanitation Assistance				1.621 (-1.105 – 4.346)
R-squared	0.323	0.377	0.395	0.481

***p < 0.01, **p < 0.05, *p < 0.1, Data presented are adjusted beta coefficients and (95% Confidence Intervals).

^+^Model D additionally adjusted for pre-tsunami living conditions (type of roof, source of water) and satisfaction with community infrastructure (school, health clinic, water and electric services, mosque facility, and roads).

Because our main interest was in identifying *modifiable* factors predicting positive outcomes (i.e., post-disaster interventions and experiences), we examined the role of displacement, as well as relief and development assistance received by the household. In this model, we found that for each month of continued displacement the durable solutions index score decreased (β = -0.11, *P* < 0.001) after adjusting for other factors. The receipt of food assistance was negatively associated with the overall index score (β = -8.56, *P* = 0.01). The receipt of shelter assistance as well as legal assistance had approximately the same magnitude of effect on the index score, reflecting a 2.70 (*P* = 0.0067) and 2.36 point increase in the score out of 100 (*P* < 0.001), respectively.

In order to understand how the domains of the durable solutions total score were associated with the predictor covariates, we repeated the multivariate linear regression analysis on each of the component durable solutions scales, focusing on modifiable risk factors (Table 4). Households’ current standard of living was significantly positively associated with the receipt of shelter assistance (β = 4.22, *P* = 0.001), legal assistance (β = 2.15, *P* = 0.024), and provision of water for sanitation and hygiene (β = 3.29, *P* = 0.049). Prolonged displacement duration was associated with a 1.7-point decrease in the standard of living score for each year of displacement (*P* < 0.001). Perceived safety and security in the community was not significantly associated with the provision of any type of humanitarian assistance, but was negatively associated with prolonged displacement duration (β = -0.197, *P* = 0.005). Participation in public affairs was significantly associated with the receipt of livelihood assistance (β = 2.156, *P* = 0.031) and highly associated with the receipt of food assistance (β = 42.58, *P* < 0.001). Restitution of property was only significantly associated with receipt of water for hygiene and sanitation (β = -3.89, *P* = 0.021). Access to livelihoods and employment was significantly positively associated with the receipt of legal assistance (β = 1.43, *P* = 0.032), but paradoxically negatively associated with the receipt of livelihood assistance (β = -1.71, *P* = 0.040).

**Table 4:** **Durable solutions scales by indicator**

	Standard of living	Safety and security	Access to livelihoods	Participation in public affairs	Restitution of property
**Pre-Tsunami Conditions**					
Household Size	0.312 (–0.142 – 0.766)	0.245 (-0.492 – 0.982)	-0.680*** (-1.019 – 0.341)	-0.092 (-0.67– 0.486)	-0.138 (-0.687 – 0.411)
Education of Head of Household					
Less than Primary	REF	REF	REF	REF	REF
Primary	0.823 (-2.255 – 3.902)	-0.546 (-3.159 – 2.067)	0.585 (-3.726 – 4.896)	1.613 (-3.175 – 6.401)	0.462 (-2.465 – 3.388)
Secondary	1.108 (-1.777 – 3.992)	-1.194 (-4.757 – 2.370)	2.092 (-2.356 – 6.540)	2.432 (-1.969 – 6.833)	1.586 (-0.825 – 3.997)
High School	1.621 (-1.494 – 4.735)	-1.348 (-3.358 – 0.661)	3.839* (-0.510 – 8.189)	2.186 (-2.377 – 6.750)	0.241 (-2.744 – 3.226)
College or University	5.807*** (2.619 – 8.995)	-0.685 (-3.320 – 1.950)	8.818*** (4.632 – 13.00)	2.938 (-1.813 – 7.690)	2.077 (-1.025 – 5.179)
Number of Household Members Employed	0.423 (-1.018 – 1.865)	0.185 (-0.795 – 1.166)	1.742*** (0.561 – 2.923)	1.088 (-0.291 – 2.467)	-1.348 (-3.069 – 0.373)
Employment Status of Head of Household	2.11 (-3.432 – 7.653)	-12.15*** (-19.59 – –4.719)	4.552* (-0.229 – 9.333)	-8.403*** (-13.970 – 2.837)	2.337 (-2.872 – 7.545)
Home Owned	-1.182 (-5.140 – 2.776)	-2.318 (-5.809 – 1.173)	1.054 (-1.465 – 3.573)	2.117 (-2.330 – 6.564)	12.19** (0.798 – 23.58)
Monthly Income ($10 USD)	0.157*** (0.0520 – 0.262)	0.091* (-0.0057 – 0.188)	0.349*** (0.264 – 0.434)	-0.060 (-0.152 – 0.032)	0.047 (-0.021 – 0.115)
**Tsunami Damages**					
Distance of Home from Coast	-0.00092 (-0.0116 – 0.0098)	-0.00025 (-0.0102 – 0.097)	-0.0027 (-0.0149 – 0.0096)	-0.0127* (-0.028 – 0.002)	0.0016 (-0.005 – 0.008)
Number of Tsunami-Related Deaths in Household	-0.376 (-1.005 – 0.253)	-0.459 (-1.129 – 0.211)	-0.784*** (-1.316 – 0.251)	-0.310 (-1.153 – 0.534)	-0.0225 (-0.745 – 0.700)
Tsunami–Related Damage					
No Damage	REF	REF	REF	REF	REF
Minor Damage	-1.493 (-3.619 – 0.634)	-1.853 (-5.200 – 1.493)	-1.852 (-5.671 – 1.967)	1.422 (-2.610 – 5.454)	-1.008 (-4.496 – 2.481)
Major Damage	-3.442* (-7.057 – 0.173)	-1.687 (-5.007 – 1.634)	-1.318 (-5.630 – 2.993)	2.609 (-2.450 – 7.668)	-1.112 (-4.349 – 2.125)
Destroyed Completely	-4.902*** (-8.436 – 1.369)	4.248** (0.602 – 7.894)	-1.594 (-6.512 – 3.323)	0.736 (-3.806 – 5.277)	-3.218* (-6.840 – 0.404)
**Displacement**					
Living in Same Village	-3.233* (-6.892 – 0.426)	3.708* (-0.709 – 8.126)	-2.090* (-4.496 – 0.315)	-0.926 (-6.751 – 4.900)	8.602* (-0.314 – 17.52)
Total Displacement Duration (months)	-0.139*** (-0.207 – 0.0719)	-0.197*** (-0.328 – 0.067)	-0.0388 (-0.116 – 0.0386)	0.048 (-0.057 – 0.153)	0.029 (-0.0798 – 0.138)
Ever Displaced to a Barrack	0.833 (-1.316 – 2.982)	0.403 (-1.092 – 1.899)	-0.0817 (-1.353 – 1.189)	-1.073 (-3.433 – 1.286)	1.152 (-0.679 – 2.984)
Ever Stayed with a Host Family	1.374 (-0.364 – 3.113)	-0.458 (-2.303 – 1.386)	-0.16 (-1.776 – 1.455)	-0.185 (-2.281 – 1.912)	0.303 (-1.879 – 2.485)
**Assistance Received**					
Received Shelter Assistance	4.218*** (2.053 – 6.384)	-1.659 (-4.112 – 0.794)	-0.356 (-2.466 – 1.753)	-2.884* (-6.218 – 0.450)	1.326 (-2.124 – 4.776)
Received Livelihood Assistance	0.4 (-1.169 – 1.970)	-1.063 (-3.042 – 0.917)	-1.705** (-3.324 – –0.0860)	2.156** (0.212 – 4.100)	-1.723* (-3.775 – 0.329)
Received Food Assistance	1.675 (-4.523 – 7.872)	1.398 (-6.336 – 9.132)	-0.974 (-8.848 – 6.900)	42.58*** (33.99 – 51.17)	-1.735 (-9.017 – 5.548)
Received Drinking Water Assistance	-0.207 (-4.613 – 4.198)	-5.13 (-12.33 – 2.068)	3.409 (-0.824 – 7.643)	1.241 (-5.037 – 7.518)	2.525 (-1.627 – 6.677)
Received Water for Hygiene	3.293** (0.0227 – 6.562)	3.654 (-5.737 – 13.04)	-3.957 (-8.985 – 1.071)	2.150 (-4.454 – 8.754)	-3.489** (-6.408 – –0.570)
Received Healthcare Assistance	-2.41 (-8.146 – 3.326)	-1.475 (-6.498 – 3.549)	3.946 (-2.307 – 10.20)	-0.589 (-6.132 – 4.954)	-1.142 (-6.306 – 4.022)
Received Legal Assistance	2.153** (0.306 – 3.999)	1.422 (-0.729 – 3.573)	1.434** (0.138 – 2.730)	-1.882* (-4.114 – 0.349)	1.055 (-0.759 – 2.869)
Received Sanitation Assistance	0.225 (-4.240 – 4.690)	-2.316 (-5.822 – 1.190)	1.073 (-2.036 – 4.182)	-1.969 (-6.387 – 2.449)	-0.483 (-3.678 – 2.712)

***p < 0.01, **p < 0.05, *p < 0.1, Data presented are adjusted beta coefficients and (95% Confidence Intervals).

All models additionally adjusted for pre-tsunami living conditions (type of roof, source of water) and satisfaction with community infrastructure (school, health clinic, water and electric services, mosque facility, and roads).

### Livelihoods

Because access to livelihoods and employment seemed to decrease as a result of the receipt of livelihood assistance, we sought to determine predictors of either increased or decreased income (after adjusting for inflation) comparing pre-tsunami conditions to those in 2011. We dichotomized the change in income as 1 if it was the same or increased after the tsunami (n = 164) or 0 if it was lower than before the tsunami (n = 436), and performed multiple logistic regression analysis (Table 5).

**Table 5:** **Logistic regression for same or better income now versus pre-tsunami**

	Unadjusted odds ratio		Adjusted odds ratio	
	OR (95% CI)	***P***	OR (95% CI)	***P***
**Pre-Tsunami Conditions**				
Household Size	0.948 (0.842 – 1.068)	0.366	1.122 (0.948 – 1.328)	0.169
Head of Household Employed	0.552 (0.101 – 3.016)	0.477	0.836 (0.164 – 4.264)	0.822
Home Owned	1.132 (0.572 – 2.238)	0.711	1.318 (0.661 – 2.631)	0.416
Monthly Household Income (per $10 USD)	0.928 (0.884 – 0.973)	0.004	0.908 (0.875 – 0.943)	<0.001
**Tsunami Damages and Displacement**				
Household Tsunami Damage				
No Damage	1	REF	1	REF
Minor Damage (Habitable)	0.87 (0.414 – 1.828)	0.701	1.741 (0.295 – 10.27)	0.524
Major Damage (Uninhabitable)	0.320 (0.141 – 0.728)	0.009	0.707 (0.117 – 4.260)	0.693
Destroyed Completely	0.653 (0.311 – 1.371)	0.246	1.357 (0.227 – 8.117)	0.727
Household Tsunami–Related Deaths	0.851 (0.726 – 0.997)	0.046	0.767 (0.620 – 0.949)	0.017
Total Displacement Duration (in months)	0.989 (0.969 – 1.011)	0.313	0.985 (0.959 – 1.011)	0.244
Ever Displaced to Barrack	0.85 (0.537 – 1.346)	0.472	1.392 (0.872 – 2.221)	0.157
Ever Displaced to Host Family	1.338 (0.927 – 1.930)	0.114	1.715 (1.192 – 2.467)	0.005
Residing in Village of Origin	1.592 (0.694 – 3.654)	0.259	1.579 (0.491 – 5.079)	0.427
**Receipt of Livelihood Assistance**				
By 1–2 months	0.884 (0.582 – 1.343)	0.548	1.082 (0.622 – 1.881)	0.771
By 1 year	0.507 (0.358 – 0.717)	<0.001	0.515 (0.315 – 0.843)	0.01
By 6 years	1.834 (0.685 – 4.912)	0.215	3.021 (1.368 – 6.670)	0.008

We also examined the time of receipt of livelihood assistance in three categories: received within 1–2 months of the tsunami, received within 1 year of the tsunami (by the end of 2005), or received between 1 year and 6 years after the tsunami. In this model, we find that households with higher pre-tsunami income were more likely to have their income decrease after the tsunami (adjusted OR 0.91, *P* < 0.001). Income was also likely to decrease with a greater number of tsunami-related deaths (adjusted OR 0.767, *P* = 0.017), and was significantly likely to increase if the household was displaced to a host family rather than a barrack or camp during their post-tsunami displacement experience (adjusted OR 1.72, *P* = 0.005). In this analysis we found that the timing of the receipt of livelihood assistance was a significant factor. Whereas receipt of livelihood assistance very shortly after the tsunami (within one year) significantly lowered the odds of a household attaining or surpassing its pre-tsunami monthly income (adjusted OR 0.52, *P* = 0.010) the odds of having a higher income increased threefold (the highest magnitude of any covariate) for those households who received livelihood assistance between 1 and 6 years after the tsunami (adjusted OR 3.02, *P* = 0.008). When we added the timing of the receipt of humanitarian assistance to the livelihoods PCA score, we found that there was no longer a significant positive or negative association between receipt of livelihood assistance and access to livelihoods (data not shown). In an effort to further characterize the role of timing the receipt of livelihood assistance, we analyzed predictors of receipt of livelihood assistance at 1–2 months, within one year, and by six years (Additional file 1: Table S1). Here we found that receiving assistance immediately was negatively associated with having one’s home completely destroyed (adjusted OR 0.157, *P* = 0.020). Receiving assistance within the first year was predicted by higher pre-tsunami income levels (adjusted OR 1.024, *P* = 0.027) and by residing in barracks (adjusted OR 1.521, *P* = 0.011). There was a perfect correlation of failing to receive assistance between one and six years predicted by not owning a home, residing in a different village than prior to the tsunami, and having a head of household unemployed before the tsunami. There was a trend toward significance for residing in host families (rather than barracks) predicting receiving livelihood assistance between one and six years (adjusted OR 2.440, *P* = 0.053). These findings suggest that markers of higher pre-tsunami socioeconomic status were significantly associated with the receipt of livelihood assistance, and that living in host families rather than barracks was likely beneficial in the receipt of livelihood assistance during the longer-term development process.

### The attainment of durable solutions

The attainment of durable solutions for internally displaced persons requires not only absolute measures of a population, but also relative ones, to determine whether or not those integrated elsewhere have achieved the same level of standard of living, safety and security, livelihood access, participation in public affairs, and restitution of property relative to those who returned to their villages of origin (without discrimination). To make this determination, we performed Wald tests comparing those who returned to their villages of origin to those who integrated elsewhere, and found that attainment of durable solutions was equivalent in the two groups (Table 6).

**Table 6:** **Comparing durable solutions for return status**

	Durable solutions total index	Standard of living	Safety and security	Livelihood	Participation in public affairs	Restitution of property
**Returned Home**	100.1	99.94	100.2	99.96	100.03	101.3
	(97.63 – 102.5)	(97.43 – 102.4)	(99.55 – 100.8)	(98.13 – 101.8)	(99.16 – 100.89)	(100.5 – 102.1)
**Integrated Elsewhere**	99.37	100.9	97.95	100.5	99.97	85.88
	(95.43 – 103.3)	(96.64 – 105.1)	(92.76 – 103.1)	(97.48 – 103.5)	(96.00 – 103.94)	(82.57 – 89.19)
Wald Test *P*	0.781	0.711	0.387	0.755	0.976	<0.001

All scores have a mean equal to 100 with standard deviation of 10.

There were no significant differences in the total durable solutions index (100.1 for those returned home v 99.37 for those integrated elsewhere, *P* = 0.781), or for any of the individual indicator scores with the exception of restitution of property (101.3 for those returned to their villages of origin versus 85.88 for those who integrated elsewhere, *P* < 0.001). Restitution of property however is not comparable in the two groups, however, because it derives not only from home ownership, but also from return to the same plot of land as before the tsunami which is inherently precluded for those who integrated elsewhere. All other measures reflect the successful and non-discriminatory achievement of durable solutions for those who integrated elsewhere in Aceh Province.

## Discussion

In this cluster sample survey of two districts of Aceh, Indonesia, we found that there was a significant improvement in housing quality and satisfaction with infrastructure services after post-tsunami reconstruction, whereas measures of household income significantly decreased. Further, we found that equivalent levels of durable solutions were attained by both those who returned to their villages of origin and those who integrated elsewhere in the province. Those who did not return to their villages of origin were primarily those who did not own homes prior to the tsunami. The primary aim of this analysis, however, was to determine and understand modifiable factors (those that occurred after the disaster and during the recovery phase) predicting better household outcomes, such that they may inform future policy and program decisions after a major natural disaster. Using principal component analysis and multivariate regression, we found that the receipt of shelter assistance and legal assistance consistently predicted better outcomes for households. There was a less clear role for livelihood assistance, although we present evidence to suggest that the timing of livelihood assistance was a critical determinant on whether or not it resulted in a positive long-term household impact, wherein its provision after one year of tsunami significantly increased the odds of a household retaining or increasing its monthly income. The most consistent negative modifiable predictor of successful durable solutions outcomes was the total duration of displacement – the longer families were displaced, the poorer their standard of living, safety and security, and participation in public affairs measured six years after the disaster.

Shelter assistance provided after the tsunami, including the reconstruction of destroyed homes was significantly positively associated with improved standard of living and the total durable solutions score, suggesting that the Indonesian government’s shelter program (administered by the BRR) was successful in its policy of “building back better”. Although Sheppard & Hill [22] found that the provision of shelter assistance in the emergency setting was significantly associated with an improvement in household income and livelihoods due to “backward” (from the production of the shelter itself) and “forward” (due to household productivity in the economy) linkages, we were unable to replicate this finding. This may be a result of the fact that although their models adjusted for the household size and age of head of household, they did not adjust for other types of humanitarian assistance received. In our study, we found that those who received livelihood assistance were significantly more likely to receive shelter assistance as well – an association which may have differentiated our findings.

Although measures of livelihood access decreased slightly when compared to the pre-tsunami period, access to livelihoods was equivalent for both returnees and those who had integrated elsewhere. In a series of models, we found that timing of livelihood assistance was significantly associated with changes in household income, wherein assistance later in the recovery cycle predicted higher incomes in the long-term but was not significantly associated with the attainment of durable solutions. Types of livelihood assistance offered after the tsunami included cash for work programs [23] as well as the titling of land parcels and the provision of an estimated 142,544 microcredit loans [2], although the effectiveness of these programs at the end of the results chain has not been assessed. The UNORC reported that a “bubble economy” was generated in the wake of the disaster, but warned that the “artificial influx of capital and employment can generate a reliance that is difficult to end without careful planning and a commensurate economic development program”. Unemployment in Aceh increased by 2008 as recovery efforts came to a halt and was estimated at 9%, suggesting that short-term recovery programs may not have a sustainable impact on household livelihoods. Here we also found that the populations receiving livelihood assistance at various stages of the development cycle varied. Those receiving assistance within the first year (those who experienced a decrease in monthly income) were more likely to live in barracks rather than host families and have higher pre-tsunami incomes, whereas those receiving livelihood assistance later on were more likely to have owned homes and had a head of household that was employed prior to the tsunami, and lived in host families rather than barracks or camps after the tsunami.

The duration of displacement was the most significant negative predictor of the attainment of durable solutions. From both a practical and policy perspective, this is one measure that may be of particular interest to humanitarian actors, as displacement itself is known to be associated with poorer mental health outcomes as well [24, 25] or may be a proxy for other daily stressors [26]. In our study, there was a wide range of time during which households were displaced with a mean of 20.72 months and a standard deviation of 11.39 months. We have found that after adjusting for home ownership and income in this population, displacement duration was significantly reduced in families who lived with host families rather than government barracks immediately following tsunami (Robinson et al., manuscript forthcoming). In tandem with the benefit in post-tsunami income, these findings suggest that there are benefits to living in host families rather than government- and NGO-supported encampments following the disaster.

While Harrell-Bond once noted that “humanitarian work… is thought to be selfless, motivated by compassion, and by its very definition suggests good work…[but] it is not expected that anyone (most especially the recipients) should examine the quality or quantity of what is given” [27], the field is now under intense scrutiny. Even so, few published works have assessed causal associations between humanitarian assistance provision and rights-based outcomes for its beneficiaries. Beneficiary satisfaction at the household level also distinguishes this assessment from the Tsunami Recovery Impact Assessment and Monitoring System (TRIAMS), which relies primarily on government-reported infrastructure indicators [2]. Furthermore, this assessment performed at the end of the results chain, with fewer than 0.1% of the population internally displaced, allows for an assessment of long term and sustained outcomes recognizing that “the scope for finding durable solutions to displacement is critically influenced by the political economy conditions” [28].

The *Framework on Durable Solutions for Internally Displaced Persons* as an international norm addresses what many authors have pointed out as disparate or narrow perspectives on assessing humanitarian aid, which has made modeling of impact and recovery extremely difficult [29]. Whereas Arlikatti et al. [8] sought to address this issue with the use of a modified domestic assets approach (DAI), the *Framework* has two distinct advantages in its comprehensive scope (beyond asset measurement) as well as self-weighting for conceptually equal indicator scores.

There are several limitations to our study. The first is due to its cross-sectional nature. Because we did not track the same households from prior to the tsunami forward, our establishment of a temporal baseline was contingent on participant recall. Despite the lack of longitudinal data, we found that recall of household characteristics was generally consistent throughout three study rounds, the first of which was conducted in 2005. In their review of impact evaluation methodologies, ALNAP provides that “impact can be assessed despite the absence of pre-existing data [using] retrospective and rolling baselines” [4]. Accordingly, the largest known longitudinal study initiated prior to the tsunami in Aceh was similarly unable to provide a baseline for community and facility infrastructures, and rather used retrospective information from households and communities, although the restrospective nature of the data should be considered when results are difficult to interpret [30].

A second limitation of our study is the non-probability sampling methodology used in order to locate households who had moved away from their original villages of origin, which were the sampling clusters. Although our analyses were adjusted for the design effect generated by clustering and stratification, the true inflation of variance due to the non-probability sample cannot be determined. Furthermore, there may be clustering effects decreasing variance within the referring households and the snowball-sampled households, including the possibility that snowball-sampled households may have stronger social networks than those that were not referred. Although this type of problem was both unavoidable and anticipated, we do not feel that it compromises the validity of the results because our intention was to capture households from the same village of origin (which would maintain the intraclass correlation) and we do not have significant reason to suspect that recall of pre-tsunami conditions would significantly differ amongst the two groups. A final limitation in the data is our inability to make distinctions between specific programmatic activities that the participating households received, although we do believe that in a broad and comprehensive assessment, this sectoral analysis provides a rich source of data.

## Conclusion

We provide a novel analytic approach and conceptual framework for use in disaster research, as well as the identification of predictors for the attainment of durable solutions. This framework can also be implemented in a household survey format in scenarios wherein longitudinal studies are not feasible due to the sudden onset of disaster. We found that the strongest modifiable variable associated with long-term positive changes in household outcomes was the duration of displacement, which could be significantly reduced by agencies facilitating movement to host families rather than relocation sites in the recovery process. Study evidence also suggests that after the disaster recovery period has ended, livelihood programs as well as shelter and legal assistance are effective in increasing longer-term access to livelihoods, as well as improving conditions of living and participation in public affairs. Current and future humanitarian responses may benefit not only from the identification of a comprehensive set of impact indicators, but also from a greater wealth of studies examining modifiable factors related to improved durable solutions.

## Electronic supplementary material

Additional file 1: **Predictors of Receipt of Livelihood Assistance at Different Stages of the Recovery Cycle.** (XLS 29 KB)

## References

[1] USAID (2005). Fact Sheet #38.

[2] UNORC (2008). Tsunami Recovery Indicator Package.

[3] Masyrafah H, McKeon JM (2008). Post-Tsunami aid Effectiveness in Aceh: Proliferation and Coordination in Reconstruction. Brookings Global Economy and Development.

[4] Proudlock K, Ramalingam B, Sandison P (2009). Improving Humanitarian Impact Assessment: Bridging Theory and Practice. 8th Review of Humanitarian Action: Performance, Impact and Innovation.

[5] Mollica RF, McInnes K, Sarajlic N, Lavelle J, Sarajlic I, Massagli MP (1999). Disability associated with psychiatric comorbidity and health status in Bosnian refugees living in Croatia. JAMA.

[6] Miller KE, Rasmussen A (2010). War exposure, daily stressors, and mental health in conflict and post-conflict settings: bridging the divide between trauma-focused and psychosocial frameworks. Soc Sci Med.

[7] Bolton P, Bass J, Murray L, Lee K, Weiss W, McDonnell SM (2007). Expanding the scope of humanitarian program evaluation. Prehosp Disaster Med.

[8] Arlikatti S, Peacock WG, Prater CS, Grover H, Sekar AS (2010). Assessing the impact of the Indian Ocean tsunami on households: a modified domestic assets index approach. Disasters.

[9] Martin S (2005). Challenge of Finding Solutions for a Growing Population of Forced Migrants. The Uprooted: Improving Humanitarian Responses to Forced Migration.

[10] OECD (2010). Glossary of key Terms in Evaluation and Results Based Management.

[11] UN OCHA: *Joint Humanitarian Impact Evaluation: Options Paper*. New York: Evaluation and Studies Section, United Nations Office for the Coordination of Humanitarian Affairs; [https://ochanet.unocha.org/p/Documents/Joint_Humanitarian_IE_Options_Paper_FINAL_BECK_OCHA.pdf]

[12] IASC (2010). Framework on Durable Solutions for Internally Displaced Persons.

[13] OCHA (2004). Guiding Principles on Internal Displacement.

[14] Lopes Cardozo B, Bilukha OO, Gotway Crawford CA, Shaikh I, Wolfe MI, Gerber ML, Anderson M (2004). Mental health, social functioning, and disability in postwar Afghanistan. JAMA.

[15] Irmansyah I, Dharmono S, Maramis A, Minas H (2010). Determinants of psychological morbidity in survivors of the earthquake and tsunami in Aceh and Nias. Int J Ment Health Syst.

[16] Chambers R, Conway G (1992). Sustainable Rural Livelihoods: Practical Concepts for the 21st Century. IDS Discussion Paper.

[17] Oppenheim-Mason K, Narayan D (2005). Measuring women’s Empowerment: Learning from Cross-National Research. Measuring Empowerment: Cross-Disciplinary Perspectives.

[18] Filmer D, Pritchett LH (2001). Estimating wealth effects without expenditure data-or tears: an application to educational enrollment in states of India. Demography.

[19] Vyas S, Kumaranayake L (2006). Constructing socio-economic status indices: how to use principal components analysis. Health Policy Plan.

[20] Gwatkin DE, Rutstein S, Johnson K, Suliman E, Wagstaff A, Amouzou A (2007). Socio-Economic Differences in Health, Nutrition, and Population: Brazil. Country Reports on HNP and Poverty.

[21] Pomeroy RS, Pollnac RB, Katon BM, Predo CD (1997). Evaluating factors contributing to the success of community-based coastal resource management: the Central Visayas Regional Project-1, Phillipines. Ocean Coast Manag.

[22] Hill R, Sheppard S (2005). The Economic Impact of Shelter Assistance in Post-Disaster Settings.

[23] Doocy S, Gabriel M, Collins S, Robinson C, Stevenson P (2006). Implementing cash for work programmes in post-tsunami Aceh: experiences and lessons learned. Disasters.

[24] Lee CT, Du YB, Christina D, O’Rourke EJ, Palfrey JS, Belfer ML (2014). Displacement as a predictor of functional impairment in tsunami-exposed children. Disasters.

[25] Roberts B, Ocaka KF, Browne J, Oyok T, Sondorp E (2008). Factors associated with post-traumatic stress disorder and depression amongst internally displaced persons in northern Uganda. BMC Psychiatry.

[26] Fernando GA, Miller KE, Berger DE (2010). Growing pains: the impact of disaster-related and daily stressors on the psychological and psychosocial functioning of youth in Sri Lanka. Child Dev.

[27] Harrell-Bond BE (1986). Imposing aid: Emergency Assistance to Refugees.

[28] Harlid N, Christensen A (2010). The Development Challenge of Finding Durable Solutions for Refugees and Internally Displaced People.

[29] Peacock W, Dash N, Zhang Y, Rodriguez H, Quarantelli E, Dynes R (2006). Shelter and Housing Recovery Following Disaster. The Handbook of Disaster Research.

[30] Frankenberg E, Friedman J, Saadah F, Sikoki B, Suriastini W, Sumantri C, Thomas D, Amin S, Das J, Goldstein M (2008). Assessment of Health and Education Services in the Aftermath of a Disaster. Are you Being Served? New Tools for Measuring Service Delivery.

[CR31] The pre-publication history for this paper can be accessed here:http://www.biomedcentral.com/1471-2458/14/1168/prepub

